# Meta-analysis of diagnostic accuracy of nucleic acid amplification tests for abdominal tuberculosis

**DOI:** 10.1371/journal.pone.0289336

**Published:** 2023-11-27

**Authors:** Yanqin Shen, Likui Fang, Bo Ye, Guocan Yu

**Affiliations:** Zhejiang Tuberculosis Diagnosis and Treatment Center, Affiliated Hangzhou Chest Hospital, School of Medicine, Zhejiang Chinese Medicine and Western Medicine Integrated Hospital, Zhejiang University, Hangzhou, Zhejiang, China; New Delhi Tuberculosis Centre, INDIA

## Abstract

**Background:**

Abdominal tuberculosis (TB) is a severe extrapulmonary TB, which can lead to serious complications. Early diagnosis and treatment are very important for the prognosis and the diagnosis of abdominal TB is still difficult.

**Methods:**

We searched PubMed, the Cochrane Library, Embase, China National Knowledge Infrastructure, and the Wanfang database for studies evaluating the diagnostic accuracy of NAATs for abdominal TB until August 2020. Any types of study design with full text were sought and included. The risk of bias was assessed using the Quality Assessment of Diagnostic Accuracy Studies tool. Subgroup analysis, meta-regression analysis and sensitivity analysis were used to explore the sources of heterogeneity. Stata version 15.0 with the *midas* command packages was used to carry out meta-analyses.

**Results:**

We included a total of 78 independent studies from 53 articles; 64 with CRS as the reference standard, and 14 with culture as the reference standard. The pooled sensitivity, specificity, and the areas under summary receiver operating characteristic (SROC) curves (AUC) were 58% (51%–64%; I^2^ = 87%), 99% (97%–99%; I^2^ = 81%), and 0.92 (0.89–0.94) compared with CRS, respectively. The pooled sensitivity, specificity, and the AUC values of the SROC were 80% (66%–90%; I^2^ = 56%), 96% (92%–98%; I^2^ = 84%), and 0.97 (0.95–0.98) compared with culture, respectively. The heterogeneity of sensitivity and specificity was significant.

**Conclusions:**

NAATs had excellent efficacy in the diagnosis of abdominal TB regardless of the reference standard and regardless of the subtype of abdominal TB. Multiplex PCR with multiple target genes may improve diagnostic sensitivity, and stool specimens may also be used for the diagnosis of abdominal TB in addition to tissue and ascites.

## Introduction

Tuberculosis (TB) is a serious threat to global health [1]. Severe types of extrapulmonary tuberculosis (EPTB) increase tuberculosis-related mortality, especially in immunodeficient populations. Abdominal TB is a common form of EPTB caused by *Mycobacterium TB* (MTB) infection of the abdominal organs, mainly including intestinal and peritoneal TB [2]. Abdominal TB can cause many complications, such as intestinal obstruction, intestinal perforation, which seriously affect the quality of life and prognosis of patients [3]. Therefore, early diagnosis and treatment of abdominal TB is very important to reduce the incidence of serious abdominal complications. Crohn’s disease (CD), inflammatory bowel disease (IBD) and abdominal TB have similar clinical presentations and pathologies [4]. It is easy to misdiagnose abdominal TB as CD and IBD, thus delaying the treatment. The diagnosis of abdominal TB is still challenging.

Nucleic acid amplification tests (NAATs) play a huge role in the diagnosis of microbiological infections, making it faster and more accurate [5]. NAATs are widely used in the diagnosis of TB, which make the early diagnosis of TB possible [6, 7]. In the diagnosis of EPTB, NAATs are also fast, accurate and efficient, and they improve the detection rate of TB, especially in specimens with low bacterial content, such as tuberculous lymphadenitis and tuberculous meningitis [8, 9]. Abdominal TB is a type of paucibacillary EPTB and NAATs also have these advantages in its diagnosis. However, the diagnostic efficacy of NAATs for abdominal TB remains controversial. The aim of this systematic review and meta-analysis is to assess the diagnostic validity of NAATs for the diagnosis of abdominal TB.

## Methods

### Design and registration

We conducted a systematic review and meta-analysis of diagnostic test accuracy to assess the diagnostic efficacy of NAATs for abdominal TB. We have registered the protocol on the International Platform of Registered Systematic Review and Meta-analysis Protocols (INPLASY), and the registration number is INPLASY202060030 [10]. The Preferred Reporting Items for Systematic Reviews and Meta-Analysis (PRISMA) 2020 statement was followed for reporting our systematic review [11].

### Information sources

PubMed, the Cochrane Library, Embase, China National Knowledge Infrastructure (CNKI), and the Wanfang database were searched for studies that evaluate NAAT’s diagnostic accuracy for abdominal TB until August 2020.

### Search strategy

The search strategies were conducted by Yanqin Shen and Likui Fang.

There was no language restriction on our search. Search strategy of PubMed was as follows:

#1 "Tuberculosis, Gastrointestinal"[Mesh] OR "Gastrointestinal Tuberculosis" OR “Intestinal tuberculosis" OR "Peritonitis, Tuberculous"[Mesh] OR "Tuberculosis, Peritoneal" OR "peritoneal tuberculosis" OR "Tuberculous ascites" OR "Tuberculous Peritonitis" OR "Abdominal TB" OR "intra-Abdominal TB"

#2 ("Nucleic Acid Amplification Techniques"[Mesh] OR "Polymerase Chain Reaction"[Mesh] OR "Real-Time Polymerase Chain Reaction"[Mesh] OR "Reverse Transcriptase Polymerase Chain Reaction"[Mesh] OR "Multiplex Polymerase Chain Reaction"[Mesh] OR "genexpert"[tw] OR Xpert OR "genotype"[tw])

#3 #1 AND #2

Similar search formulae were used for Embase, the Cochrane Library, CNKI, and Wanfang databases.

### Eligibility criteria

#### Type of study

Any types of studies can be included, such as retrospective studies, prospective studies, case-control studies. We included original researches with full text that assessed the diagnostic accuracy of NAATs for abdominal TB. The reference standard should be appropriate and precisely defined in the study. True positive (TP), false positive (FP), false negative (FN), and true negative (TN) values were provided directly in the articles or contain necessary data to calculate these values. We excluded articles reported in languages other than Chinese and English, case reports, studies with a specimen size of less than 10, conference coverages, and studies with abstracts but no full text.

#### Patients

We included studies, which contain patients diagnosed with abdominal TB through NAATs. We had no restrictions on age, gender, and nations.

#### Index tests

NAATs were considered as index test.

#### Reference standards

Bacteriological confirmation of MTB (positive culture of MTB and/or microscopic identification of acid‐fast bacilli on stained specimen smear) was reference gold standard.

Composite reference standard (CRS): Radiological characteristics (such as tree-in-bud pattern and cavity) and histopathological features of the suspected tissue specimen (features of chronic granulomatous inflammation with caseous necrosis/ caseating granuloma). Positive of reference standard test and/or positive of all CRS mentioned were considered abdominal TB. If all factors were negative, it was considered as non- abdominal TB.

#### Literature screening and selection

Primary search results matching the search strategy were imported into the ENDNOTE X9.2 literature management software. Two investigators (Yanqin Shen and Likui Fang) screened candidate studies independently by reviewing the titles and abstracts followed by the full text. Disagreements between two the researchers were resolved by discussion with a third researcher (Guocan Yu).

### Data extraction

Name of first author; year of publication; country of study; reference standard; TP, FP, FN, and TN values of the test; method of patient selection; test method; NAAT target genes; subtypes of abdominal TB (such as intestinal and peritoneal TB); type of specimen; specimen processing procedures (e.g., homogenization) and specimen condition along with other parameters were extracted. One article that simultaneously reported the accuracy of different specimen types, different reference standards, or different NAAT target genes for abdominal TB diagnosis was considered as to include separate studies based on different specimen types, or different reference standards, or different NAAT target genes [12]. The same two researchers independently extracted relevant data from each included study and cross-check their respective information. Disagreements between two the researchers were resolved by discussion with a third researcher, similar to that used during the literature selection phase.

### Quality evaluation

The two researchers assessed the quality of the relevant literature using a revised tool for Quality Assessment of Diagnostic Accuracy Studies (QUADAS-2) [13] independently for the different reference standards and the disagreements between researchers were solved by discussion with a third researcher (Guocan Yu). According to the PRISMA-DTA statement, systematic review and meta-analysis of diagnostic test accuracy studies was not required to assess publication bias. The strength of the body of evidence was assessed using The Grading of Recommendations Assessment, Development and Evaluation (GRADE) guideline.

### Data synthesis and statistical analysis

TP, FP, FN, and TN values were obtained from each included study, and the estimated pooled sensitivity and specificity of NAAT for abdominal TB associated with the 95% confidence interval (CI) were calculated against culture or CRS, using bivariate random-effects models. We generated forest plots for sensitivity and specificity for each study and calculate the areas under summary receiver operating characteristic (SROC) curves (AUC). We assessed heterogeneity between studies using *I*^2^ statistics. An *I*^2^ value of 0% was indicative of no heterogeneity, while a value greater than 50% indicated significant heterogeneity [14]. Subgroup analyses were done to evaluate the diagnostic accuracy of NAATs for abdominal TB, such as different test methods, subtypes of abdominal TB, types of specimens, methods of patient selection (consecutive or convenience), methods of decontamination (with or without N-acetyl-L-cysteine/sodium hydroxide [NALC-NaOH]), conditions of sample (frozen or fresh), homogenization methods (mechanical or otherwise). If the heterogeneity was obvious, meta-regression analyses and sensitivity analysis were used to explore the source of heterogeneity. The meta-analysis for predefined variable types were performed using at least four published studies. We analyzed the data from studies against CRS and culture separately. We used Stata version 15.0 (Stata Corp., College Station, TX, USA) with the *midas* command packages to generate forest plots of sensitivity and specificity with 95% CI and carry out meta-analyses and meta-regression analyses.

## Results

### Identification of studies and study characteristics

**Fig 1:**
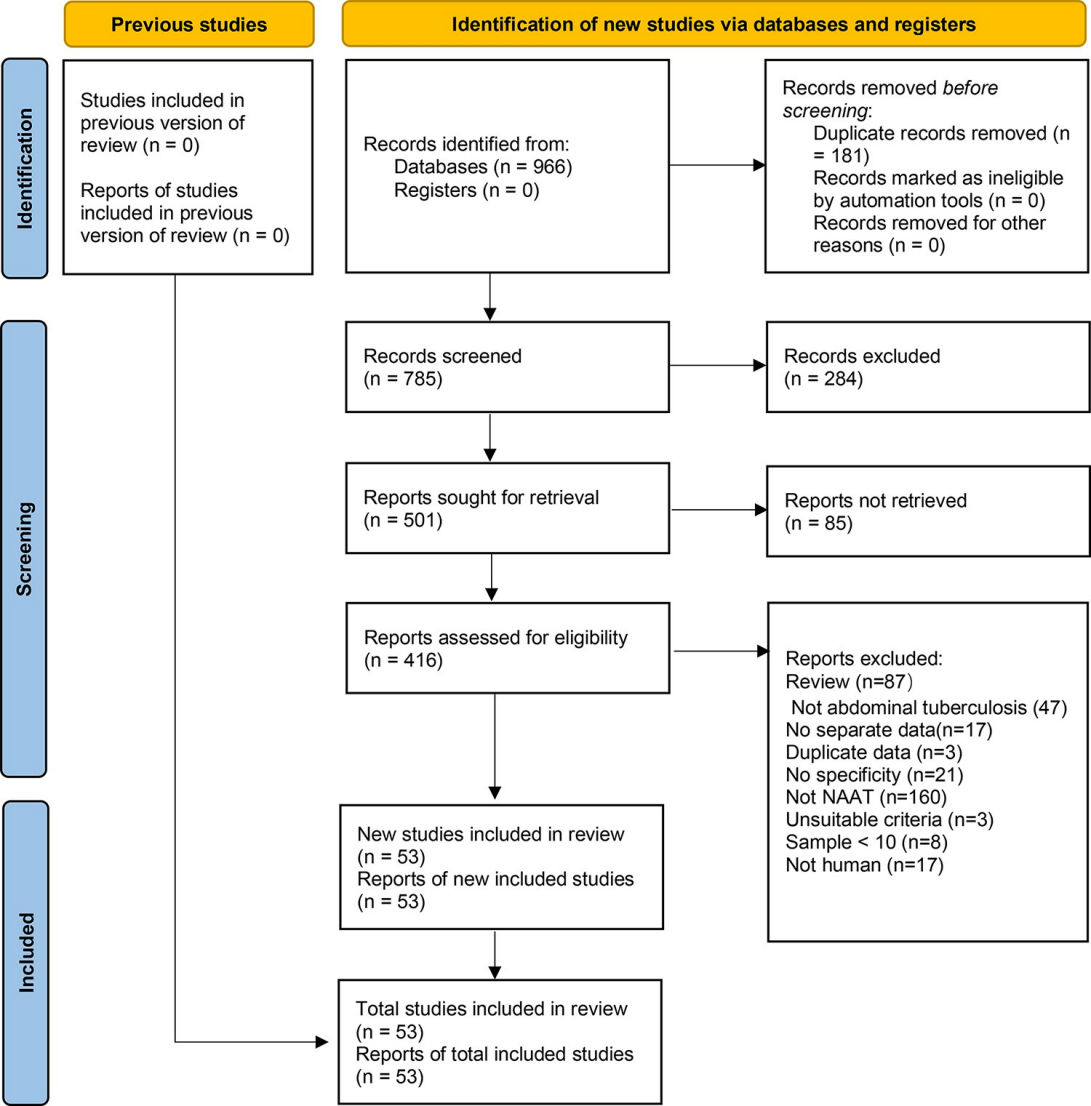
PRISMA flow chart of literature retrieval. In total, 104, 31, 429, 171, and 231 articles were found in PubMed, the Cochrane Library, Embase, China National Knowledge Infrastructure, and the Wanfang database, respectively. NAAT: nucleic acid amplification test.

By searching the relevant databases using our predefined search strategies, we found 966 candidate articles. By eliminating duplicates followed by the screening of titles, abstracts, and full texts, 53 articles met the inclusion criteria and were included in this study for meta-analyses [2, 4 12, 15–64]. The PRISMA flow chart of literature retrieval was shown in Fig 1. Fifteen articles were published in Chinese [16–19, 21, 24, 27, 29, 30, 38, 42, 43, 51, 59, 64], and the remaining 38 articles were published in English. The kappa index of agreement value between the two researchers for selection and data extraction was 0.735 (95% CI, 0.621–0.937). Eleven articles published in languages other than Chinese and English were excluded. We excluded 21 articles that reported sensitivity only (no specificity values were listed) and 17 articles did not report separate abdominal TB data. Three articles used the same data from the included articles so we also excluded them [65–67]. Three articles used reference standards other than those defined in this study, one article used genome sequencing as the reference standard [68], and the other two used histopathology data as the reference standard [69, 70]; as these did not meet the inclusion criteria of this study, they were excluded. The types of specimens used in the included articles were ascites fluid, stool, and tissue, abdominal TB types were mainly peritoneal and intestinal TB, and NAAT target genes were mainly IS6110, ropB, and MPB64. According to the principles defined in the methodology section, we included a total of 78 independent studies; 64 with CRS as the reference standard, and 14 with culture as the reference standard. Table 1 demonstrates all the included study characteristics. When CRS was used as the reference standard, a total of 4383 specimens were included in 64 studies, with specimen sizes ranging from 13 to 191 with a mean specimen size of 68.5. When culture was used as the reference standard, a total of 740 specimens were included in the 14 studies. Specimen sizes ranged from 10 to 139 with a mean specimen size of 52.9.

**Table 1:** Characteristics of the included studies.

Author	Year	County	Sample type	Reference	ATB type	Target gene	Test method	N	TP	FP	FN	TN	Type of research	Sample condition	Homogenisation	Decontamination	Patient selection method
Gan, H.	1995	China	Tissue	CRS	Intestinal TB	IS6110	PCR	83	27	2	9	45	Case-control	Paraffin-embedded and fresh	Unreport	Deparaffinized	Convenience
Jiang, H.	1997	China	Ascitic fluid	CRS	Peritoneal TB	Unreport	PCR	112	20	3	38	51	Case-control	Fresh	Unreport	Unreport	Convenience
Wu, Z.	1997	China	Ascitic fluid	CRS	Peritoneal TB	Unreport	PCR	43	10	3	10	20	Prospective	Fresh	Unreport	Unreport	Convenience
Hao, Z.	1998	China	Ascitic fluid	CRS	Peritoneal TB	Unreport	PCR	51	11	3	10	27	Case-control	Fresh	Unreport	Unreport	Convenience
Liu, W.	1998	China	Ascitic fluid	CRS	Peritoneal TB	Unreport	PCR	48	18	1	12	17	Case-control	Fresh	Unreport	Unreport	Convenience
Gan, H.	2002	China	Tissue	CRS	Intestinal TB	IS6110	PCR	69	25	0	14	30	Case-control	Paraffin-embedded and fresh	Unreport	Deparaffinized	Convenience
Huang, W.	2003	China	Tissue	CRS	Intestinal TB	Unreport	PCR	29	17	2	5	5	Case-control	Fresh	Unreport	Unreport	Convenience
Amarapurkar, DN.	2004	India	Tissue	CRS	Intestinal TB	IS6110	PCR	80	13	1	47	19	Case-control	Paraffin-embedded	Unreport	NALC-NaOH	Convenience
Balamurugan, R.	2006	India	Stool	CRS	Intestinal TB	IS6110	PCR	48	16	0	2	30	Case-control	Fresh	Mechanical	NALC-NaOH	Consecutive
Yang, J.	2007	China	Ascitic fluid	CRS	Peritoneal TB	Unreport	PCR	74	26	1	11	36	Case-control	Fresh	Unreport	Unreport	Convenience
Bandyopadhyay, D.	2008	India	Ascitic fluid	CRS	Peritoneal TB	IS6110	PCR	50	15	4	4	27	Case-control	Fresh	Unreport	NALC-NaOH	Convenience
Pulimood, A. B.	2008	India	Tissue	CRS	Intestinal TB	Unreport	PCR	40	5	1	15	19	Case-control	Paraffin-embedded	Unreport	Deparaffinized	Convenience
Ma, Z.	2008	China	Ascitic fluid	CRS	Peritoneal TB	Unreport	PCR	76	30	1	16	29	Case-control	Fresh	Unreport	Unreport	Convenience
Kim, S. H.a	2009	Korea	Ascitic fluid	CRS	Peritoneal TB	Unreport	PCR	20	5	0	6	9	Prospective	Fresh	Unreport	Unreport	Consecutive
Kim, S. H.b	2009	Korea	Tissue	CRS	Peritoneal TB	Unreport	PCR	18	6	0	6	6	Prospective	Fresh	Unreport	Unreport	Consecutive
Gu, Q.	2009	China	Tissue	CRS	Intestinal TB	IS6110	FQ-PCR	72	23	6	13	30	Case-control	Paraffin-embedded and fresh	Unreport	Deparaffinized	Convenience
Han, M.	2009	China	Tissue	CRS	Intestinal TB	IS6110	PCR	47	3	3	23	18	Case-control	Fresh	Unreport	Unreport	Convenience
Jin, X. J.a	2010	Korea	Tissue	Culture	Intestinal TB	IS6110	PCR	19	8	0	1	10	Case-control	Paraffin-embedded	Unreport	Deparaffinized	Convenience
Jin, X. J.b	2010	Korea	Tissue	CRS	Intestinal TB	IS6110	PCR	97	20	0	35	42	Case-control	Paraffin-embedded	Unreport	Deparaffinized	Convenience
Mishra, P. K.a	2010	India	Tissue	Culture	Intestinal TB	16S rRNA	Real-time PCR	52	27	0	1	24	Case-control	Paraffin-embedded	Unreport	Deparaffinized	Convenience
Mishra, P. K.b	2010	India	Tissue	CRS	Intestinal TB	16S rRNA	Real-time PCR	102	45	0	33	24	Case-control	Paraffin-embedded	Unreport	Deparaffinized	Convenience
Vadwai, V.	2011	India	Ascitic fluid	CRS	Peritoneal TB	rpoB	Xpert	13	3	0	0	10	Prospective	Fresh	Mechanical	NALC-NaOH	Consecutive
Vinaykumar, Hallur.a	2013	India	Ascitic fluid	CRS	Peritoneal TB	IS6110	PCR	87	23	0	14	50	Prospective	Fresh	Mechanical	NALC-NaOH	Convenience
Vinaykumar, Hallur.b	2013	India	Ascitic fluid	CRS	Peritoneal TB	16S rRNA	PCR	87	13	0	24	50	Prospective	Fresh	Mechanical	NALC-NaOH	Convenience
Vinaykumar, Hallur.c	2013	India	Ascitic fluid	CRS	Peritoneal TB	devR	PCR	87	24	0	13	50	Prospective	Fresh	Mechanical	NALC-NaOH	Convenience
Vinaykumar, Hallur.d	2013	India	Ascitic fluid	CRS	Peritoneal TB	IS6110 or 16S rRNA or devR	Multiplex PCR	87	28	0	9	50	Prospective	Fresh	Mechanical	NALC-NaOH	Convenience
Vinaykumar, Hallur.e	2013	India	Tissue	CRS	Intestinal TB	IS6110	PCR	96	31	2	9	54	Prospective	Fresh	Mechanical	NALC-NaOH	Convenience
Vinaykumar, Hallur.f	2013	India	Tissue	CRS	Intestinal TB	16S rRNA	PCR	96	24	0	16	56	Prospective	Fresh	Mechanical	NALC-NaOH	Convenience
Vinaykumar, Hallur.g	2013	India	Tissue	CRS	Intestinal TB	devR	PCR	96	32	2	8	54	Prospective	Fresh	Mechanical	NALC-NaOH	Convenience
Vinaykumar, Hallur.h	2013	India	Tissue	CRS	Intestinal TB	IS6110 or 16S rRNA or devR	Multiplex PCR	96	35	2	5	54	Prospective	Fresh	Mechanical	NALC-NaOH	Convenience
Lei, Y.	2013	China	Tissue	CRS	Intestinal TB	IS6110	PCR	176	46	7	34	89	Case-control	Paraffin-embedded and fresh	Unreport	Deparaffinized	Convenience
Sharma, K.a	2013	India	Tissue	CRS	Intestinal TB	IS6110	PCR	70	28	0	12	30	Case-control	Fresh	Mechanical	NALC-NaOH	Convenience
Sharma, K.b	2013	India	Tissue	CRS	Intestinal TB	MPB64	PCR	70	30	0	10	30	Case-control	Fresh	Mechanical	NALC-NaOH	Convenience
Sharma, K.c	2013	India	Tissue	CRS	Intestinal TB	IS6110 or MPB64	Multiplex PCR	70	31	0	9	30	Case-control	Fresh	Mechanical	NALC-NaOH	Convenience
Zmak, L.	2013	Croatia	Ascitic fluid	Culture	Peritoneal TB	rpoB	Xpert	10	1	0	2	7	Retrospective	Fresh	Manual	NALC-NaOH	Convenience
Chen, Y.	2013	China	Tissue	CRS	Intestinal TB	IS6110	PCR	50	8	0	17	25	Case-control	Paraffin-embedded	Manual	Deparaffinized	Convenience
Fei, B. Y.a	2014	China	Tissue	CRS	Intestinal TB	Unreport	FQ-PCR	65	16	2	13	34	Case-control	Frozen	Unreport	Unreport	Consecutive
Fei, B. Y.b	2014	China	Stool	CRS	Intestinal TB	Unreport	FQ-PCR	65	24	3	5	33	Case-control	Frozen	Unreport	Unreport	Consecutive
Scott, L. E.	2014	South Africa	Ascitic fluid	Culture	Peritoneal TB	rpoB	Xpert	139	23	3	16	97	Retrospective	Fresh	Unreport	NALC-NaOH	Convenience
Bera, C.	2015	India	Ascitic fluid	CRS	Peritoneal TB	rpoB	Xpert	28	4	0	17	7	Prospective	Fresh	Unreport	Unreport	Convenience
Katsunori Sekine.a	2015	Japan	Tissue	CRS	Intestinal TB	Unreport	PCR	35	7	0	21	7	Retrospective	Fresh	Unreport	Unreport	Convenience
Katsunori Sekine.b	2015	Japan	Stool	CRS	Intestinal TB	Unreport	PCR	24	3	0	10	11	Retrospective	Fresh	Unreport	Unreport	Convenience
Katsunori Sekine.c	2015	Japan	Intestinal fluid	CRS	Intestinal TB	Unreport	PCR	24	7	0	13	4	Retrospective	Fresh	Unreport	Unreport	Convenience
Yadav, S. K.	2015	India	Ascitic fluid	CRS	Peritoneal TB	IS6110	PCR	87	29	0	4	54	Prospective	Fresh	Unreport	Unreport	Consecutive
Yang, B.a	2015	China	Ascitic fluid	CRS	Peritoneal TB	Unreport	Real-time PCR	16	4	1	9	2	Case-control	Fresh	Unreport	Unreport	Convenience
Yang, B.b	2015	China	Ascitic fluid	CRS	Peritoneal TB	IS1081	LAMP	16	7	2	6	1	Case-control	Fresh	Unreport	Unreport	Convenience
Yang, J.	2016	China	Tissue	CRS	Intestinal TB	rpoB	Xpert	80	19	0	13	48	Case-control	Fresh	Unreport	Unreport	Convenience
Kumar, S.	2017	India	Tissue	CRS	Intestinal TB	rpoB	Xpert	98	3	0	34	61	Case-control	Fresh	Manual	Unreport	Consecutive
Li, Y.	2017	China	Ascitic fluid	Culture	Peritoneal TB	rpoB	Xpert	54	3	2	1	48	Prospective	Fresh	Mechanical	Unreport	Consecutive
Rufai, S. B.a	2017	India	Ascitic fluid	Culture	Peritoneal TB	rpoB	Xpert	67	12	0	5	50	Prospective	Fresh	Unreport	NALC-NaOH	Consecutive
Rufai, S. B.b	2017	India	Ascitic fluid	Culture	Peritoneal TB	hsp‑65 and esat‑6	Multiplex PCR	67	9	0	8	50	Prospective	Fresh	Unreport	NALC-NaOH	Consecutive
Ullah, I.	2017	Pakistan	Ascitic fluid	Culture	Peritoneal TB	rpoB	Xpert	56	4	4	0	48	Prospective	Fresh	Manual	NALC-NaOH	Consecutive
Patel, B.	2018	India	Tissue	CRS	Intestinal TB	Unreport	PCR	107	49	8	20	30	Prospective	Fresh	Unreport	Unreport	Unreport
Fei, G.	2018	China	Ascitic fluid	CRS	Peritoneal TB	Unreport	PCR	163	5	0	22	136	Retrospective	Fresh	Unreport	Unreport	Convenience
Rakotoarivelo, R.a	2018	Madagascar	Ascitic fluid	CRS	Peritoneal TB	rpoB	Xpert	62	11	0	16	35	Prospective	Fresh	Unreport	Unreport	Consecutive
Rakotoarivelo, R.b	2018	Madagascar	Ascitic fluid	Culture	Peritoneal TB	rpoB	Xpert	42	9	2	5	26	Prospective	Fresh	Unreport	Unreport	Consecutive
Luo, F.	2018	China	Tissue	CRS	Intestinal TB	IS6110	PCR	170	14	7	35	114	Case-control	Paraffin-embedded	Unreport	Deparaffinized	Convenience
Allahyartorkaman, M.	2019	Iran	Ascitic fluid	Culture	Peritoneal TB	rpoB	Xpert	36	3	0	0	33	Cross-sectional	Fresh	Unreport	Unreport	Convenience
Bellam, B. L.	2019	India	Tissue	CRS	Intestinal TB	rpoB	Xpert	40	8	0	17	15	Retrospective	Fresh	Unreport	Unreport	Consecutive
Dahale, A. S.	2019	India	Tissue	CRS	Peritoneal TB	rpoB	Xpert	36	17	0	11	8	Case-control	Fresh	Unreport	Unreport	Convenience
Malik, S.a	2019	India	Tissue	CRS	Intestinal TB	IS6110	PCR	50	22	0	10	18	Prospective	Fresh	Unreport	Unreport	Convenience
Malik, S.b	2019	India	Tissue	CRS	Intestinal TB	MPB64	PCR	50	26	0	6	18	Prospective	Fresh	Unreport	Unreport	Convenience
Malik, S.c	2019	India	Tissue	CRS	Intestinal TB	Protein b	PCR	50	24	0	8	18	Prospective	Fresh	Unreport	Unreport	Convenience
Malik, S.d	2019	India	Tissue	CRS	Intestinal TB	Protein b or MPB64 or Protein b	Multiplex PCR	50	28	0	4	18	Prospective	Fresh	Unreport	Unreport	Convenience
Mbuh, T. P.	2019	Cameroon	Ascitic fluid	Culture	Peritoneal TB	rpoB	Xpert	13	4	1	0	8	Cross-sectional	Fresh	Unreport	NALC-NaOH	Convenience
Tadesse, M.a	2019	Ethiopia	Ascitic fluid	Culture	Peritoneal TB	rpoB	Xpert	78	5	4	2	67	Prospective	Fresh	Unreport	NALC-NaOH	Consecutive
Tadesse, M.b	2019	Ethiopia	Ascitic fluid	CRS	Peritoneal TB	rpoB	Xpert	52	7	0	15	30	Prospective	Fresh	Unreport	NALC-NaOH	Consecutive
Talib, A.	2019	Pakistan	Stool	CRS	Intestinal TB	rpoB	Xpert	100	9	11	14	66	Prospective	Frozen	Unreport	Unreport	Consecutive
Wang, Q.	2019	China	Ascitic fluid	Culture	Peritoneal TB	rpoB	Xpert	32	8	1	3	20	Retrospective	Fresh	Unreport	Unreport	Convenience
Liu, R.	2020	China	Ascitic fluid	CRS	Peritoneal TB	rpoB	Xpert	191	21	0	94	76	Retrospective	Fresh	Unreport	NALC-NaOH	Convenience
Lowbridge, C.a	2020	Malaysia	Tissue	CRS	Abdominal TB	rpoB	Xpert	57	22	0	1	34	Prospective	Fresh	Unreport	Unreport	Convenience
Lowbridge, C.b	2020	Malaysia	Tissue	CRS	Abdominal TB	Unreport	PCR	29	7	0	7	15	Prospective	Fresh	Unreport	Unreport	Convenience
Salman, A. E.	2020	Iraq	Ascitic fluid	Culture	Peritoneal TB	IS6110	PCR	75	11	17	0	47	Prospective	Frozen	Unreport	Unreport	Consecutive
Sharma, M.a	2020	India	Tissue	CRS	Abdominal TB	IS6110	PCR	65	24	0	11	30	Prospective	Frozen	Unreport	NALC-NaOH	Convenience
Sharma, M.b	2020	India	Tissue	CRS	Abdominal TB	IS6110	LAMP	65	26	0	9	30	Prospective	Frozen	Unreport	NALC-NaOH	Convenience
Sharma, M.c	2020	India	Tissue	CRS	Abdominal TB	MPB64	LAMP	65	29	0	6	30	Prospective	Frozen	Unreport	NALC-NaOH	Convenience
Sharma, M.d	2020	India	Tissue	CRS	Abdominal TB	IS6110 or MPB64	Multiplex LAMP	65	30	0	5	30	Prospective	Frozen	Unreport	NALC-NaOH	Convenience
Song, J.	2020	China	Tissue	CRS	Intestinal TB	rpoB	Xpert	38	12	0	5	21	Retrospective	Fresh	Unreport	Unreport	Convenience

ATB, abdominal tuberculosis; CRS, composite reference standard; TP, true-positive; FP, false-positive; FN, false-negative; TN, true-negative; PCR, polymerase chain reaction; FQ-PCR,fluorescent quantitative polymerase chain reaction; LAMP, loop-mediated isothermal amplification; NALC-NaOH, N-acetyl-L-cysteine/sodium hydroxide.

### Study quality

**Fig 2:**
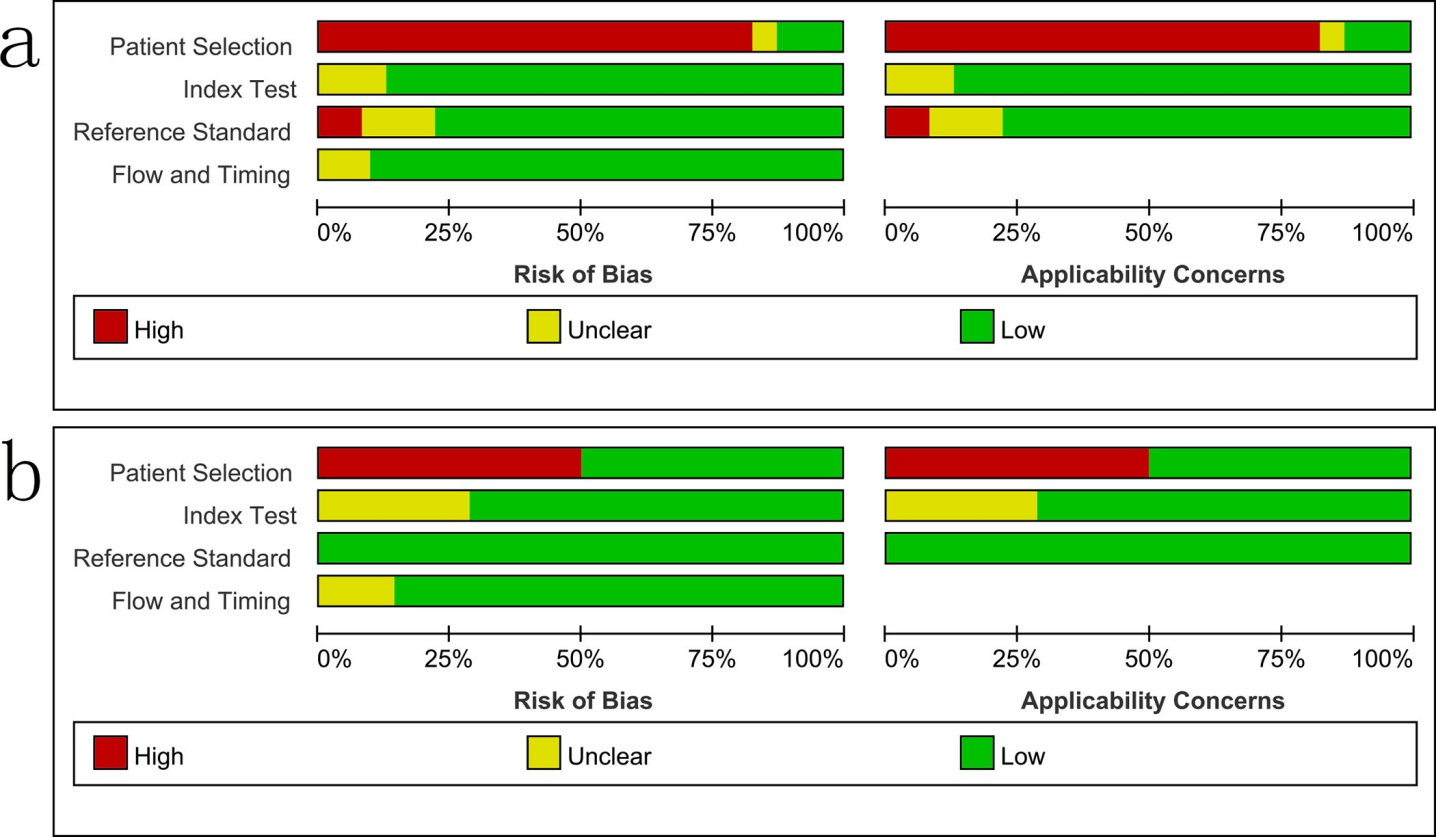
Methodological quality graphs (risk of bias and applicability concerns) across the included studies. a) composite reference standard as gold standard. b) culture as gold standard.

Fig 2 shows the results of the methodological evaluation on the quality of the included studies comparing CRS and culture. Most of the included studies used the nonconsecutive patient selection method. In the CRS reference standard, some studies did not include the anti-TB treatment response, and some studies included the results of the index test. These were the major sources of bias. The risk of bias that originated from the two aspects of the index test and the flow and timing were relatively low. According to the GRADE guidelines, the quality of evidence of this meta-analysis was high, and the recommendation level was moderate when CRS was used as the gold standard. The quality of evidence of this meta-analysis was high, and the recommendation level was strong when culture was used as the gold standard.

### Diagnostic accuracy of NAATs for abdominal TB

**Fig 3:**
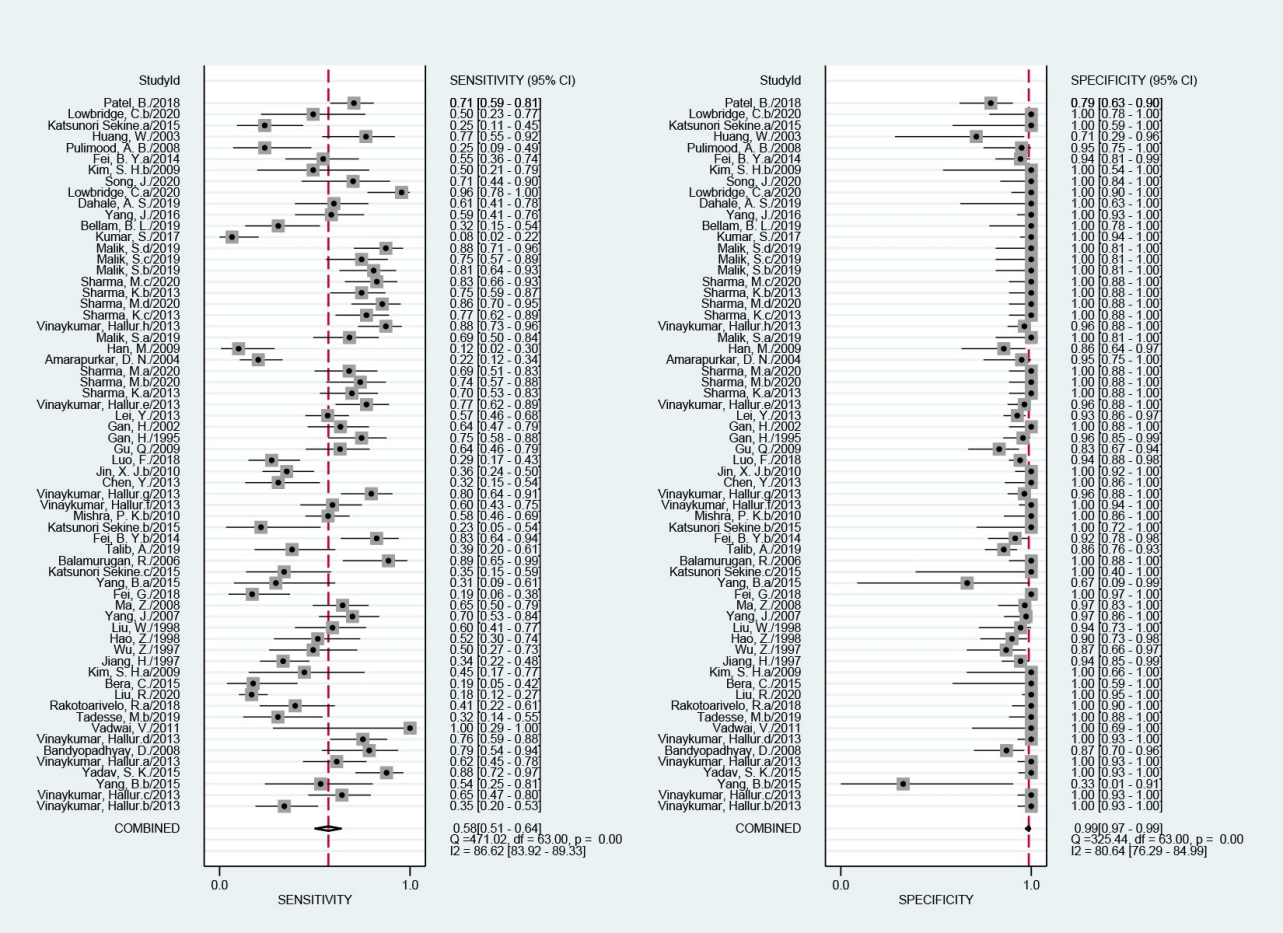
Forest plot for the sensitivity and specificity of NAATs for the diagnosis of abdominal TB compared with a composite reference standard.

**Fig 4:**
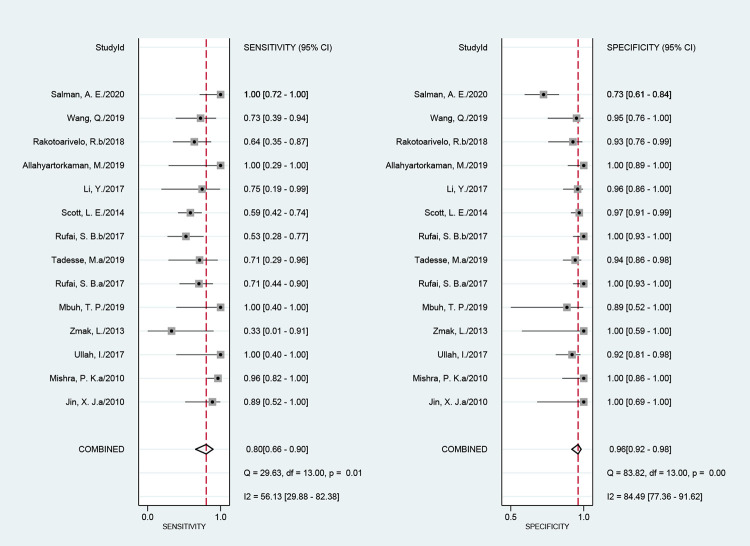
Forest plot for the sensitivity and specificity of NAATs for the diagnosis of abdominal TB compared with culture.

The efficacy of NAATs for the diagnosis of abdominal TB was assessed in 64 studies when CRS was the reference standard. The pooled sensitivity, specificity, and the area under the curve (AUC) of the SROC were 58% (51%–64%; I^2^ = 87%), 99% (97%–99%; I^2^ = 81%), and 0.92 (0.89–0.94), respectively (Fig 3). The heterogeneity of sensitivity and specificity was significant. Fourteen studies assessed the efficacy of NAATs for abdominal TB compared with culture. The pooled sensitivity, specificity, and the AUC values of the SROC were 80% (66%–90%; I^2^ = 56%), 96% (92%–98%; I^2^ = 84%), and 0.97 (0.95–0.98), respectively (Fig 4). The heterogeneity of sensitivity and specificity was also significant.

### Subgroup analyses

Subgroup analysis of parameters for which sufficient data were available. The analyzed results of each subgroup are listed in Table 2 (compared with CRS). The results show that the heterogeneity in sensitivity and specificity remained high significant in the majority of subgroups. The heterogeneity of sensitivity and specificity in the diagnosis of abdominal TB based on the use of multiplex polymerase chain reaction (PCR) alone was insignificant. The heterogeneity in sensitivity for the remaining subgroups was significant. The heterogeneity of the specificity of the retrospective study, paraffin-embedded samples, and mechanical method for homogenization was nonsignificant, while the heterogeneity of the specificity of the remaining subgroups was highly significant. Table 3 demonstrates the analyzed results of each subgroup (sufficient data) compared with culture. For subgroups with limited data, subgroup analysis was dropped. The heterogeneity of sensitivity was insignificant except in the convenience patient selection method group. Additionally, the heterogeneity of specificity in most subgroups was insignificant.

**Table 2:** Subgroup analysis for different parameters with sufficient data compared with a composite reference standard.

Parameters		No. of study	Sensitivity (59% CI)	I^2^	Specificity (59% CI)	I^2^	AUC (95% CI)
Test methods	PCR	39	56% (48–64%)	84%	98% (96–99%)	72%	0.91 (0.88–0.93)
	Multiplex PCR	4	82% (74–88%)	0%	99% (89–100%)	15%	0.92 (0.90–0.94)
	Xpert	12	45% (27–65%)	89%	100% (96–100%)	98%	0.95 (0.93–0.97)
Target gene	IS6110	18	60% (48–71%)	88%	98% (94–99%)	73%	0.93 (0.90–0.95)
	rpoB	12	45% (27–65%)	89%	100% (96–100%)	98%	0.95 (0.93–0.97)
Abdominal TB type	Peritoneal TB	23	51% (42–61%)	84%	99% (96–100%)	86%	0.84 (0.80–0.87)
	Intestinal TB	35	58% (48–66%)	87%	98% (95–99%)	75%	0.93 (0.90–0.95)
Specimen type	Ascitic fluid	21	51% (41–61%)	85%	99% (95–100%)	87%	0.84 (0.81–0.87)
	Tissue	38	62% (53–69%)	87%	99% (97–100%)	77%	0.94 (0.92–0.96)
	Stool	4	64% (31–87%)	88%	92% (83–97%)	77%	0.92 (0.89–0.94)
Patient selection	Consecutive	14	51% (35–67%)	85%	100% (93–100%)	84%	0.92 (0.89–0.94)
	Convenience	49	59% (52–66%)	87%	98% (97–99%)	79%	0.92 (0.89–0.94)
Decontamination	With NALC-NaOH	21	68% (58–77%)	92%	99% (97–100%)	76%	0.97 (0.95–0.98)
Sample condition	Fresh	47	58% (50–66%)	87%	99% (98–100%)	85%	0.92 (0.89–0.94)
	Frozen	7	72% (59–82%)	74%	97% (91–99%)	81%	0.94 (0.91–0.95)
	Paraffin-embedded	6	34% (24–45%)	80%	98% (90–100%)	24%	0.73 (0.69–0.77)
Homogenization	Mechanical	13	72% (64–79%)	71%	99% (97–100%)	25%	0.94 (0.92–0.96)
Type of research	Case-control	29	54% (45–63%)	85%	96% (93–98%)	73%	0.91 (0.88–0.93)
	Prospective	28	68% (60–75%)	80%	100% (97–100%)	82%	0.93 (0.91–0.95)
	Retrospective	7	29% (19–42%)	74%	100% (99–100%)	0%	1.00 (0.99–1.00)

TB, tuberculosis; PCR, polymerase chain reaction; AUC, areas under summary receiver operating characteristic curves; NALC-NaOH, N-acetyl-L-cysteine/sodium hydroxide.

**Table 3:** Subgroup analysis for different parameters with sufficient data compared with culture.

Parameters		No. of study	Sensitivity (59% CI)	I^2^	Specificity (59% CI)	I^2^	AUC (95% CI)
Test methods	Xpert	10	68% (56–78%)	0%	96% (94–97%)	0%	0.93 (0.90–0.95)
Target gene	rpoB	10	68% (56–78%)	0%	96% (94–97%)	0%	0.93 (0.90–0.95)
Abdominal TB type	Peritoneal TB	12	73% (59–84%)	32%	96% (92–98%)	82%	0.93 (0.90–0.95)
Specimen type	Ascitic fluid	12	73% (59–84%)	32%	96% (92–98%)	82%	0.93 (0.90–0.95)
Patient selection	Consecutive	7	77% (58–89%)	37%	95% (88–98%)	86%	0.93 (0.91–0.95)
	Convenience	7	83% (61–94%)	76%	98% (93–99%)	47%	0.99 (0.97–0.99)
Decontamination	With NALC-NaOH	7	67% (51–79%)	20%	97% (93–99%)	34%	0.91 (0.88–0.93)
Sample condition	Fresh	11	67% (56–76%)	0%	97% (94–98%)	11%	0.90 (0.87–0.92)
Type of research	Prospective	7	77% (58–89%)	37%	95% (88–98%)	86%	0.93 (0.91–0.95)

TB, tuberculosis; AUC, areas under summary receiver operating characteristic curves; NALC-NaOH, N-acetyl-L-cysteine/sodium hydroxide.

### Meta-regression and sensitivity analysis

Meta-regression analysis could not be performed with Stata (with the *midas* command packages) if there were more than two subgroups that used the same parameter. Studies that did not report relevant data were removed from the meta-regression analysis of the relevant parameters. When compared with CRS, methods of patient selection (consecutive or convenience) and methods of decontamination (with or without NALC-NaOH) had no effect on the sensitivity and specificity of NAATs for abdominal TB (meta-regression P > 0.05), and homogenization methods (mechanical or otherwise) may affect the sensitivity and specificity of NAATs (meta-regression P < 0.05). Compared with culture, subtypes of abdominal TB (peritoneal or intestinal TB), types of specimens (ascitic fluid or tissue), and homogenization methods, may have effects on sensitivity and specificity of NAATs (meta-regression P < 0.05). By contrast, methods of patient selection had no effect on sensitivity and specificity of NAATs (meta-regression P > 0.05).

**Fig 5:**
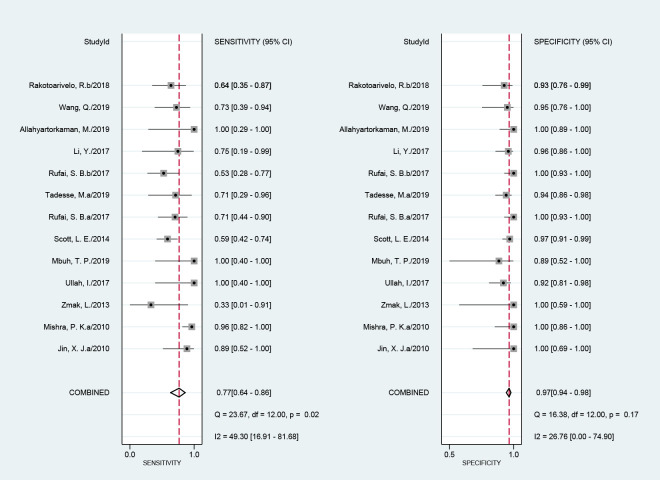
Forest plot for the sensitivity and specificity of NAATs for the diagnosis of abdominal TB after eliminating the article published by Salman et al., when compared with culture [71].

Sensitivity analysis did not identify specific articles as sources of heterogeneity in sensitivity and specificity compared with CRS. When compared with culture, the heterogeneity of both sensitivity and specificity decreased significantly after the article published by Salman et al. was eliminated. The pooled sensitivity, specificity, and the AUC value of the SROC after the article was eliminated were 77% (64%–86%, I^2^ = 49%), 97% (94%–98%, I^2^ = 27%), and 0.97 (0.95–0.98), respectively (Fig 5). The heterogeneity of sensitivity and specificity was insignificant.

## Discussion

Despite NAATs important role in the diagnosis of TB, its diagnostic ability for abdominal TB remains inconsistent. This study systematically evaluated the diagnostic efficacy of NAATs for abdominal TB using a meta-analysis method. The study’s findings suggested that NAATs can be used as means for early and rapid diagnosis of abdominal TB. However, the heterogeneity of sensitivity and specificity was significant regardless of the reference standard which made the results less credible. Satisfactory effects of NAATs have been observed in other types of TB as well [71, 72]. The overall diagnostic accuracy of NAATs in pulmonary TB is better than in abdominal TB, which correlates with the higher bacterial content of respiratory specimens compared to abdominal TB specimens (such ascites) [71]. In other types of EPTB, the diagnostic accuracy of NAATs in different types of specimens was highly variable, but overall, the results were similar to those observed in abdominal TB [72–74].

NAATs contain multiple assays, each of which may have its own characteristics, and multiple manifestations of abdominal TB which may be a source of heterogeneity among studies [75]. Different types of PCR, Xpert MTB/RIF (Xpert), and LAMP are the common types of NAATs. In this study, the numbers of studies using real-time PCR, fluorescent quantitative PCR, and LAMP assays, were limited, and subgroup analysis could not be performed compared with CRS. When compared with culture, only the studies which used the Xpert assay had sufficient numbers for subgroup analysis. Subgroup analysis showed that the sensitivity was the highest when multiplex PCR assays were used, followed by those which used PCR, and the lowest by those using Xpert assay, but the AUC was indeed the highest for Xpert compared with CRS. This may be attributed to the fact that multiplex PCR can detect multiple target genes simultaneously, thus increasing the MTB detection rate. However, the method did not improve specificity compared to Xpert, and the total diagnostic efficacy was thus still lower than that of Xpert. Heterogeneity in sensitivity and specificity was insignificant when using multiplex PCR, and the result was reliable. This result suggested that the increase of the number of target genes in the same test may improve the sensitivity of the diagnosis. When the CRS was compared in different target genes, only the studies which used the IS6110 and ropB as target genes had sufficient numbers for subgroup analysis. IS6110 is an extensively used target gene in NAATs and ropB is a specific target gene for Xpert [76, 77]. This meta-analysis revealed that the sensitivity of IS6110 was superior to that of ropB, but the specificity and AUC were slightly lower than that of ropB. Both of these two target genes were efficient for the diagnosis of abdominal TB. In comparison with culture, only ropB was available for subgroup analysis, thus suggesting that this target gene had good diagnostic efficacy for abdominal TB.

Abdominal TB has various subtypes [78]. The common ones are peritoneal and intestinal TB, and intestinal TB can occur in any part of the gastrointestinal tract [79]. The original studies cited herein did not distinguish the specific site of intestinal TB infection, so we also did not distinguish the site of intestinal TB infection, but categorized it as intestinal TB for analysis. The specimen type which corresponded to different types of abdominal TB was also different. The common specimen for peritoneal TB is ascites, but peritoneal tissue specimens can also be obtained laparoscopically. The common specimens for intestinal TB are endoscopically obtained biopsy tissue specimens, but the stool can also be used for testing. This meta-analysis showed that for different types of specimens, the greatest diagnostic efficacy of NAATs was obtained in tissue specimens followed by stool specimens. The diagnostic efficacy in ascites was the lowest. The higher bacterial content of MTB in tissue specimens compared with ascites specimens, and the higher efficacy of NAATs with tissue specimens compared with ascites, were consistent with previous studies. However, both tissue and ascites specimens need to be obtained with invasive procedures, such as laparotomy, laparoscopy, or colonoscopy. However, these procedures are associated with some risks. Furthermore, the stool specimens can be obtained very easily and noninvasively. This study demonstrated that the use of stool specimens to detect abdominal TB also had very good diagnostic efficacy, and is an excellent alternative diagnostic route in cases in which tissue or ascites cannot be obtained. However, the diagnostic efficacy of the studies which used stools were all related with intestinal TB cases, and had limited specimen sizes. The diagnostic efficacy of peritoneal TB is still unknown, and multicenter studies with large samples are needed to further evaluate it in different types of abdominal TB. NAATs were more effective in the diagnosis of intestinal TB compared with peritoneal TB. This may be related to the different specimens tested. Intestinal TB was commonly detected by biopsy tissue specimens, whereas peritoneal TB was commonly detected by ascites. This result was different from previous studies which evaluated the diagnostic efficacy of Xpert in abdominal TB [80]. Our study showed that other NAATs, such as PCR and multiplex PCR, had superior sensitivity in abdominal TB compared with Xpert, and our study included a richer set of studies. These factors contributed to the superior sensitivity of our study.

For different conditions of specimens, our subgroup analysis showed that fresh and frozen specimens had better diagnostic efficacy compared with paraffin-embedded specimens. The reason may be attributed to the fact that the paraffin-embedded specimens may have been stored for a long time and the handling of deparaffinized specimens may have had an effect on the specimens, the exact cause of which needs to be further clarified. The efficacy of fresh and frozen specimens was similar and both were excellent. Therefore, fresh or frozen specimens should be selected for testing as far as possible, and paraffin-embedded specimens should be selected only as a last resort. However, the heterogeneity among most subgroup studies was significant especially when CRS was compared. Accordingly, the results needed to be treated with caution.

We used meta-regression and sensitivity analyses to explore the sources of heterogeneity. When compared with CRS, homogenization methods (mechanical or otherwise) may have affected sensitivity and specificity of NAATs. Subgroup analyses showed decreased heterogeneity between studies with mechanical homogenization methods, especially in terms of specificity. This may be related to the fact that the mechanical homogenization method resulted in a more homogeneous MTB within the specimens. However, the effects of this method on the results and its causes needed to be further explored. When compared with culture, subtypes of abdominal TB, types of specimen and homogenization methods might have effects on sensitivity and specificity of NAATs. However, the analysis of subgroups with a sufficient number of studies revealed that heterogeneity between studies within subgroups was still highly significant, and subgroup analyses of other variables under the same parameter were not performed owing to the limited number of studies. Therefore, the effect of these factors on heterogeneity still needs to be clarified in large sample studies. Sensitivity analysis found that the article published by Salman et al. was the main source of heterogeneity when compared with culture. The pooled sensitivity, specificity, and the AUC values of the SROC after the elimination of the article were 77%, 97%, and 0.97, respectively. The heterogeneity of sensitivity and specificity was insignificant. We read the article again carefully and found that the study population was long-term peritoneal dialysis patients, which was different from other studies, and may be the source of the heterogeneity of the article. For CRS, it may constitute a source of heterogeneity. The definition of CRS in the included original studies may also be inconsistent. For example, some studies did not include clinical manifestations, some did not include the effect of antiTB treatment, and some included index tests in the CRS, all of which can generate heterogeneity.

The present study is associated with some limitations. Research omissions were unavoidable despite our best efforts to identify relevant studies. Data from some studies could not be extracted because they did not report the results we needed. Data for some subgroups were limited, especially when culture were used as the reference standard.

## Conclusions

To the best of our knowledge, this was the first diagnostic meta-analysis for the diagnostic efficacy of NAATs for abdominal TB. We found that NAATs had excellent efficacy in the diagnosis of abdominal TB regardless of the reference standard and regardless of the subtype of abdominal TB. The test efficacy was good when different types of NAATs, different target genes, and different specimen types were used. Multiplex PCR with multiple target genes may improve diagnostic sensitivity, and stool specimens may also be used for the diagnosis of abdominal TB in addition to tissue and ascites. We hope that the results of the study will help clinicians and patients to understand in depth the role of NAATs in the diagnosis of abdominal TB.

## Supporting information

S1 File Search strategies. (DOCX)

S2 File Excluded articles. (DOCX)

S3 File PRISMA DTA checklist. (DOC)

S1 Fig Methodological quality summary. a) composite reference standard as gold standard. b) culture as gold standard.
(TIF)
