# Supplementary material for: Meta-analysis of diagnostic accuracy of nucleic acid amplification tests for abdominal tuberculosis
Source: PLoS One. 2023 Nov 27;18(11):e0289336. doi: 10.1371/journal.pone.0289336 (PMC10681219; doi:10.1371/journal.pone.0289336)
Supplement: S1 File — (DOCX) [file pone.0289336.s001.docx]

PUBMED and The Cochrane Library

#1 "Tuberculosis, Gastrointestinal"[Mesh] OR "Gastrointestinal Tuberculosis" OR "Intestinal tuberculosis" OR "Peritonitis, Tuberculous"[Mesh] OR "Tuberculosis, Peritoneal" OR "peritoneal tuberculosis" OR "Tuberculous ascites" OR "Tuberculous Peritonitis" OR "abdominal tuberculosis" OR "intra-abdominal tuberculosis"

#2 "Nucleic Acid Amplification Techniques"[Mesh] OR "Polymerase Chain Reaction"[Mesh] OR "Real-Time Polymerase Chain Reaction"[Mesh] OR "Reverse Transcriptase Polymerase Chain Reaction"[Mesh] OR "Multiplex Polymerase Chain Reaction"[Mesh] OR "genexpert"[tw] OR Xpert OR "genotype"[tw]

#3 #1 AND #2

Embase

#1 'gastrointestinal tuberculosis'/exp OR 'Gastrointestinal Tuberculosis' OR 'Intestinal tuberculosis' OR 'tuberculous peritonitis'/exp OR 'Tuberculosis, Peritoneal' OR 'peritoneal tuberculosis' OR 'Tuberculous ascites' OR 'Tuberculous Peritonitis' OR 'abdominal tuberculosis' OR 'intra-abdominal tuberculosis'

#2 'nucleic acid amplification'/exp OR 'polymerase chain reaction'/exp OR 'real time polymerase chain reaction'/exp OR 'reverse transcription polymerase chain reaction'/exp OR 'multiplex polymerase chain reaction'/exp OR 'xpert'/exp OR 'genotype'/exp OR genexpert

#3 #1 AND #2

Wanfang and CNKI

(核酸扩增技术 OR 核酸检测 OR 聚合酶链反应OR PCR OR Xpert OR Genexpert) AND (胃肠道结核 OR 肠结核 OR 结核性腹膜炎 OR 腹部结核 OR 腹内结核 OR 结核性腹水)
