# Supplementary material for: Meta-analysis of diagnostic accuracy of nucleic acid amplification tests for abdominal tuberculosis
Source: PLoS One. 2023 Nov 27;18(11):e0289336. doi: 10.1371/journal.pone.0289336 (PMC10681219; doi:10.1371/journal.pone.0289336)
Supplement: S2 File — (DOCX) [file pone.0289336.s002.docx]

Languages other than Chinese and English[1-11].

Reported sensitivity only[12-32].

Did not report separate abdominal TB data[33-49].

1. Niang M, Griffet P, Hugard L, Launois P. [Diagnosis of tuberculosis by specific DNA amplification directly in samples]. Presse Med. 1992;21(10):482.

2. 三郎 宿. 日本消化器病学会雑誌. 1993;90(11):2956-9. doi: 10.11405/nisshoshi1964.90.2956.

3. 達雄 荻. 腸結核. 順天堂医学. 1999;44(4):383-7. doi: 10.14789/pjmj.44.383.

4. 寛治 大. 日本臨床外科学会雑誌. 2000;61(6):1586-90. doi: 10.3919/jjsa.61.1586.

5. 雅春 長. 喀痰, 気管支洗浄液以外の材料から結核菌が分離された症例についての検討. 感染症学雑誌. 2001;75(10):870-5. doi: 10.11150/kansenshogakuzasshi1970.75.870.

6. Tuberculosis intestinal y tuberculosis pulmonar: Resección ileal y PCR para su diagnóstico. Anales de Medicina Interna. 2004;21(7).

7. 陽代 石. 産褥期に急激な腹水貯留と血清CA125の上昇を認め悪性腫瘍と偽診された結核性腹膜炎の1症例. 産婦人科の進歩. 2004;56(1):5-9. doi: 10.11437/sanpunosinpo.56.5.

8. 野歩 大. 結核性腹膜炎治療中に続発し，粘膜下腫瘍形態を呈した胃結核の１例. 日本臨床外科学会雑誌. 2009;70(5):1347-52. doi: 10.3919/jjsa.70.1347.

9. 直也 稲. 腸結核におけるQuantiFERON<SUP>&reg;</SUP> TBの意義:QuantiFERON<SUP>&reg;</SUP> TB陰性の腸結核2症例. 日本消化器内視鏡学会雑誌. 2011;53(10):3310-6. doi: 10.11280/gee.53.3310.

10. 圭 伊. 本邦初となる乾癬に対するインフリキシマブ投与後の結核発症例. 日本皮膚科学会雑誌. 2013;123(14):3109-16. doi: 10.14924/dermatol.123.3109.

11. Lê HML, Gohy P, Schillaci A, Deltour D, Laka A, Schatt P, et al. Pulmonary ascites in a train controller. Louvain Medical. 2017;136(10):591-5.

12. Gan H, Ouyang Q, Bu H. Laboratory diagnosis of tuberculous peritonitis. Zhonghua jie he he hu xi za zhi = Zhonghua jiehe he huxi zazhi = Chinese journal of tuberculosis and respiratory diseases. 1997;20(3):149-52.

13. 甘华田, 欧阳钦, 步宏, 陈德珍, 李甘地, 李蜀华, et al. 结核性腹膜炎的实验室诊断. 中华结核和呼吸杂志. 1997(03):22-5.

14. Kim KM, Lee A, Choi KY, Lee KY, Kwak JJ. Intestinal Tuberculosis: Clinicopathologic Analysis and Diagnosis by Endoscopic Biopsy. The American Journal of Gastroenterology. 1998;93(4).

15. T M, S M, S SM, N SI. Detection of Mycobacterium tuberculosis in paraffin embedded intestinal tissue specimens by polymerase chain reaction: characterization of IS6110 element negative strains. JPMA The Journal of the Pakistan Medical Association. 1998;48(6).

16. 曹月升, 张立志, 冯志山, 刘朱梅, 王建升. 胸腹水结核杆菌DNA检测两种提取方法比较. 河北中西医结合杂志. 1999;8(5):705. doi: 10.3969/j.issn.1008-8849.1999.05.011.

17. Uzunkoy A, Harma M, Harma M. Diagnosis of abdominal tuberculosis: Experience from 11 cases and review of the literature. World Journal of Gastroenterology. 2004;10(24):3647-9. doi: 10.3748/wjg.v10.i24.3647.

18. 袁春, 李斌. 肝硬化合并结核病43例分析. 中国现代医学杂志. 2005;15(2):307-8,10. doi: 10.3969/j.issn.1005-8982.2005.02.051.

19. 蔡智群, 邝小佳, 林兆原. ATA,ADA和PCR在结核性腹膜炎的应用探讨. 临床肺科杂志. 2010;15(02):265+8.

20. 杨平, 涂福文. 不同检查方法对肠结核的诊断价值. 中国基层医药. 2010;17(5):597-8. doi: 10.3760/cma.j.issn.1008-6706.2010.05.010.

21. 薛萌. 结核性腹膜炎106例临床分析. 郑州大学; 2011.

22. Ihama Y, Hokama A, Hibiya K, Kishimoto K, Nakamoto M, Hirata T, et al. Diagnosis of intestinal tuberculosis using a monoclonal antibody to Mycobacterium tuberculosis. World Journal of Gastroenterology. 2012;18(47):6974-80. doi: 10.3748/wjg.v18.i47.6974.

23. Nieuwoudt M, Lameris R, Corcoran C, Rossouw TM, Slavik T, Plessis JD, et al. Polymerase Chain Reaction Amplifying Mycobacterial DNA from Aspirates Obtained by Endoscopic Ultrasound Allows Accurate Diagnosis of Mycobacterial Disease in HIV-Positive Patients with Abdominal Lymphadenopathy. Ultrasound in Medicine & Biology. 2014;40(9).

24. 薛佳琴. 儿童结核性腹膜炎临床特点分析. 重庆医科大学; 2015.

25. Cavalli Z, Ader F, Valour F, Saison J, Boussel L, Dumitrescu O, et al. Clinical Presentation, Diagnosis, and Bacterial Epidemiology of Peritoneal Tuberculosis in Two University Hospitals in France. Infectious Diseases and Therapy. 2016;5(2):193-9. doi: 10.1007/s40121-016-0113-2.

26. Kilic MO, Saglam C, Caner SS, Kilic Y, Icen D. Evaluation of twenty-seven patients with tuberculous peritonitis. Acta Medica Mediterranea. 2016;32(1):11-6. doi: 10.19193/0393-6384_2016_1_01.

27. 戴景涛, 卓玛, 张燕. Gene-xpert MRT/RIF在青海省三南地区耐药结核病诊断中应用. 医药前沿. 2016;6(22):78-9.

28. 樊红丽, 李健健, 高丽, 张米, 谢祺, 李正伦. 3种检测方法在HIV合并结核分枝杆菌感染诊断中的对比研究. 检验医学与临床. 2017;14(3):392-5. doi: 10.3969/j.issn.1672-9455.2017.03.031.

29. 刘玉林, 黄晓楠, 赵琪林, 向玉. 荧光定量PCR技术检测结核DNA在不同类型样本中的应用. 医学信息. 2018;31(19):163-4. doi: 10.3969/j.issn.1006-1959.2018.19.051.

30. 秦万, 王琼, 王明栋, 张娟, 徐勇, 周伟才. RNA恒温扩增法与PCR-荧光探针法在含结核菌菌量较少标本中检出率的比较. 贵州医药. 2019;43(9):1457-9. doi: 10.3969/j.issn.1000-744X.2019.09.047.

31. Gaur M, Singh A, Sharma V, Tandon G, Bothra A, Vasudeva A, et al. Diagnostic performance of non-invasive, stool-based molecular assays in patients with paucibacillary tuberculosis. Scientific reports. 2020;10(1):7102. doi: 10.1038/s41598-020-63901-z.

32. 曹探赜, 杜荣辉, 商会会, 杨澄清, 周萌, 梅春林, et al. 肠结核205例临床分析. 中华消化杂志. 2020;40(4):257-60. doi: 10.3760/cma.j.cn311367-20190722-00327.

33. Portillo-Gómez L, Morris SL, Panduro A. Rapid and efficient detection of extra-pulmonary Mycobacterium tuberculosis by PCR analysis. International Journal of Tuberculosis and Lung Disease. 2000;4(4):361-70.

34. 戴景涛, 卓玛. 结核病患者各种体液标本Xpert MTB/RIF检测结果分析. 医学理论与实践. 2018;31(4):579-80. doi: 10.19381/j.issn.1001-7585.2018.04.062.

35. 杜新生, 刘兰芳, 薛承岩, 刘怀森, 周建国. LAG-IgG与TB-DNA联合检测诊断结核性胸腹腔积液. 现代中西医结合杂志. 2000(21):2125-6.

36. 胡荣盛, 徐亚丽. 荧光定量PCR法在结核杆菌检测中的价值. 现代实用医学. 2008;20(10):767-9. doi: 10.3969/j.issn.1671-0800.2008.10.007.

37. 蒋秀琴. . 镇江医学院学报. 2000(03):184.

38. 黎永学, 府伟灵, 魏明竟. 聚合酶链反应在结核性胸膜炎和结核性腹膜炎中的应用. 陕西医学检验. 1996(02):9-10.

39. 李美霞. 聚合酶链反应在结核菌检测中的临床评价. 工企医刊. 2005(05):27.

40. 刘喆, 赵强, 张樱, 王成彬. 实时荧光定量核酸扩增技术在结核筛查诊断中 的临床应用研究. 中华医院感染学杂志. 2019;29(23):3535-8. doi: 10.11816/cn.ni.2019-190103.

41. 秦东春, 李兴武, 刘新郑, 王霞, 王红梅. PCR技术检测胸腹水中结核杆菌. 河南医科大学学报. 1997(02):85-7.

42. 孙庆. 荧光定量聚合酶链反应快速检测胸腹水抗酸杆菌的临床应用. 国际检验医学杂志. 2011;32(13):1479-80. doi: 10.3969/j.issn.1673-4130.2011.13.035.

43. 王可. 腹水TB-PCR对结核性腹膜炎的诊断价值. 中华实用中西医杂志. 2001;014(002).

44. 王龙成, 魏建华, 宋广荣, 许智芳, 贾晶晶, 王芳, et al. 三种检测方法在肺外结核病诊断中价值的探讨. 临床和实验医学杂志. 2012;11(11):881-2. doi: 10.3969/j.issn.1671-4695.2012.11.030.

45. 魏建华, 薛晓红, 熊建军, 肖慧霞, 徐麟, 郭清兰, et al. 结核分枝杆菌DNA分子生物学检测. 预防医学情报杂志. 2012;28(1):76-8.

46. 向稚丹, 方峰, 陈如, 聂兴草, 董永绥, 李革. 多重聚合酶链反应系统快速诊断结核和其他细菌感染. 实用儿科临床杂志. 2005;20(5):429-30. doi: 10.3969/j.issn.1003-515X.2005.05.022.

47. 叶菊莲, 赵龙友. PCR法对结核性胸(腹)膜炎的诊断. 浙江预防医学. 1996(04):49.

48. 张建武, 魏存乐, 吉焕英, 邢福泰, 王志刚, 李玉树. 结核PCR定量测定在结核病诊断中的应用. 北方药学. 2013(10):58-9,60.

49. 周辉, 龚志军, 钟白云, 段友斌, 唐发清, 唐银. 胸腹水脑脊液中结核杆菌的检测. 实用预防医学. 2005(01):63-4.
