# Supplementary figures and images for: Meta-analysis of diagnostic accuracy of nucleic acid amplification tests for abdominal tuberculosis
Source: PLoS One. 2023 Nov 27;18(11):e0289336. doi: 10.1371/journal.pone.0289336 (PMC10681219; doi:10.1371/journal.pone.0289336)

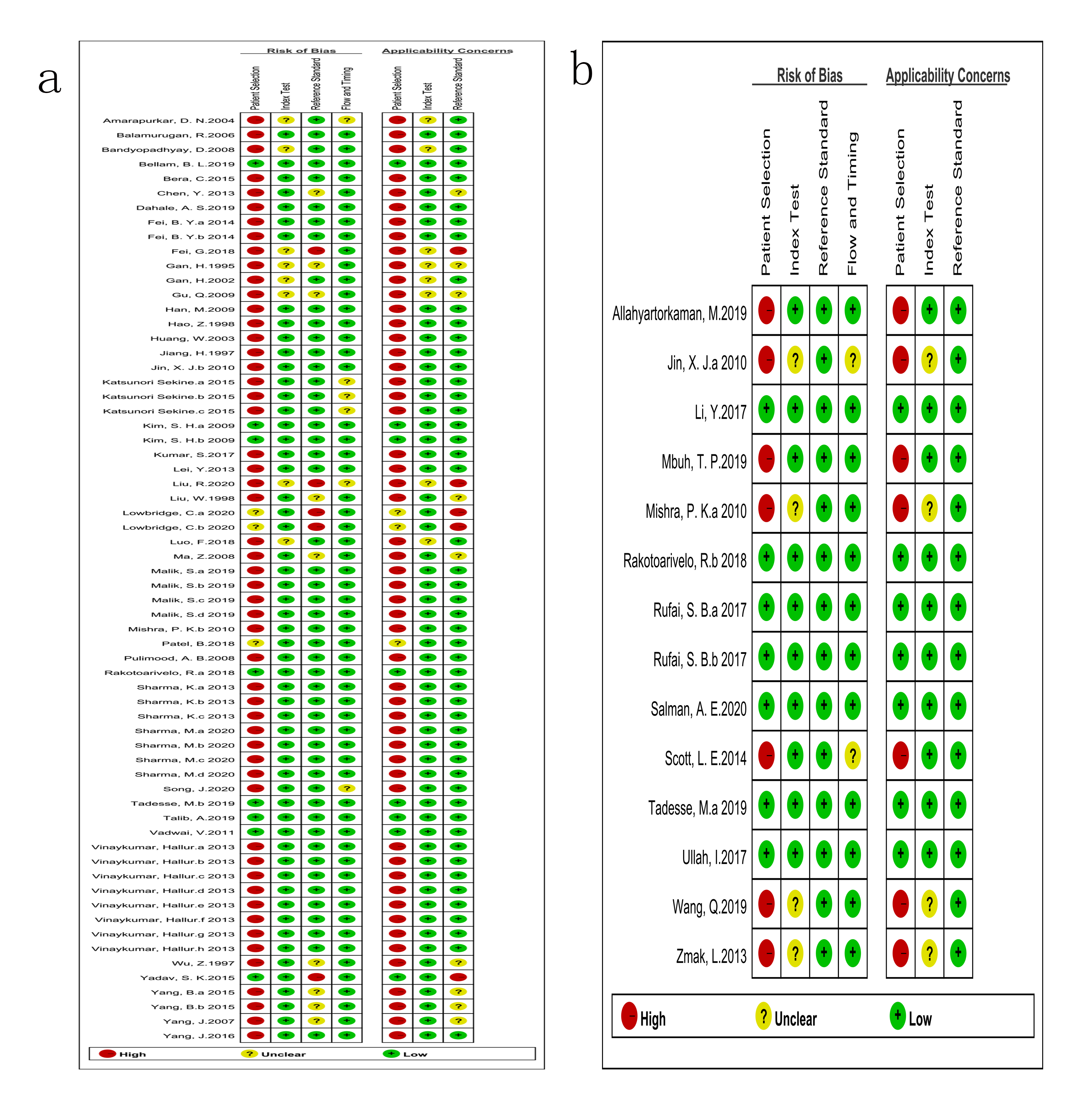

Supplement: S1 Fig — a) composite reference standard as gold standard. b) culture as gold standard. (TIF) [file pone.0289336.s004.tif]
